# Deep Recurrent Neural Networks Based Obstacle Avoidance Control for Redundant Manipulators

**DOI:** 10.3389/fnbot.2019.00047

**Published:** 2019-07-04

**Authors:** Zhihao Xu, Xuefeng Zhou, Shuai Li

**Affiliations:** ^1^Guangdong Key Laboratory of Modern Control Technology, Guangdong Institute of Intelligence Manufacturing, Guangzhou, China; ^2^School of Engineering, Swansea University, Swansea, United Kingdom

**Keywords:** recurrent neural network, redundant manipulator, obstacle avoidance, zeroing neural network, motion plan

## Abstract

Obstacle avoidance is an important subject in the control of robot manipulators, but is remains challenging for robots with redundant degrees of freedom, especially when there exist complex physical constraints. In this paper, we propose a novel controller based on deep recurrent neural networks. By abstracting robots and obstacles into critical point sets respectively, the distance between the robot and obstacles can be described in a simpler way, then the obstacle avoidance strategy is established in form of inequality constraints by general class-K functions. Using minimal-velocity-norm (MVN) scheme, the control problem is formulated as a quadratic-programming case under multiple constraints. Then a deep recurrent neural network considering system models is established to solve the QP problem online. Theoretical conduction and numerical simulations show that the controller is capable of avoiding static or dynamic obstacles, while tracking the predefined trajectories under physical constraints.

## 1. Introduction

As industrial automation develops, robot manipulators have been used in a wide range of applications such as painting, welding, assembly, etc., (Cheng et al., 2009; Yang et al., 2018a). With the evolution of intelligent manufacturing, the way robot works is also changing. In order to fulfill more difficult tasks in complex environment, the robot is required to have better execution capabilities (Pan et al., 2018). Therefore, robots with redundant DOFs have attracted much attention in the field of robotic control since its wonderful flexibility (Chan and Dubey, 1995; Zhang, 2015).

Obstacle avoidance is a core problem in the control of redundant manipulators, in order to realize human-machine collaboration and integration, robots no longer work in a separate environment that is completely isolated (Ge and Cui, 2000; Sugie et al., 2003; Lee and Buss, 2007). Instead, collaboration is required between human or other robots, as a result, the obstacle avoidance control is becoming a matter of urgency: robots need to achieve real-time avoidance of static or dynamic obstacles while completing given motion tasks.

Many obstacle avoidance methods for robot manipulators haven been reported, which are designed online or off-line. Based on stochastic sampling algorithm, a series of obstacle avoidance methods are proposed, these methods could obtain effective solutions even in ultra-redundant systems. In Wei and Ren (2018), Wei et al. propose a modified RRT based method, namely Smoothly RRT, in which a maximum curvature constraint is built to obtain a smooth curve when avoiding obstacles, simulation results also show that the method achieves faster convergence than traditional RRT based ones. In Hsu et al. (2006), Hsu discusses the probabilistic foundations of PRM based methods, a conclusion is drew that the visibility properties rather than dimensionality of C has a greater impact on the probability, and the convergence would be faster if extract partial knowledge could be introduced. However, due to the large computational costs, those methods can be hardly used online.

Different from stochastic results obtained by above mentioned methods, artificial potential field methods plan the same path each time in the same environment, which is important in industrial applications (Khatib, 1986). The basic idea of artificial potential field methods is that the target bears as an attractive pole while the obstacle creates repulsion on the robot, then the robot will be controlled to converge to the target without colliding with obstacles. At the same time, artificial potential field methods have shown great ability in tracking dynamic targets as well as avoiding dynamic obstacles. In Csiszar et al. (2011), a modified method is proposed, which describes the obstacles by different geometrical forms, both theoretical conduction and experimental tests validate the proposed method. Considering the local minimum problem that may be caused by multi-link structures, in Badawy (2016), a two minima is introduced to construct potential field, such that a dual attraction between links enables faster maneuvers comparing with traditional methods. Other improvements to artificial potential field method can be found in Tsai et al. (2001); Tsuji et al. (2002); Wen et al. (2017). Taking advantage of redundant DOFs, obstacles can be avoided by the self-motion in the null space, by calculating pseudo-inverse of Jacobian matrix, the solution can be formulated as the sum of a minimum-norm particular solution and homogeneous solutions (Cao et al., 1999; Moosavian and Papadopoulos, 2007; Krzysztof and Joanna, 2016).

The application of artificial intelligence algorithms based on neural networks provide a new idea for robotic control, these methods are considered to be very promising since its excellent learning ability (Jung and Kim, 2007). For instance, in Pan et al. (2017), the authors propose a command-filtered back-stepping method, in which a neural network based learning scheme is introduced to deal with functional uncertainties. In Pan and Yu (2017), a biomimetic hybrid controller is established, in which the control strategy consist of a feed-forward predictive machine based on a RBF Neural Network and a feedback servo machine based on a proportional-derivative controller. In Fu et al. (2018), a fuzzy logic controller is proposed for long-term navigation of quad-rotor UAV systems with input uncertainties. Experiment results show that the controller can achieve better control performance when compared to their singleton counterparts. In Fu et al. (2019), an online learning mechanism is built for visual tracking systems. The controller uses both positive and negative sample importances as input, and it is shown that the proposed weighted multiple instance learning scheme achieves wonderful tracking performance in challenging environments. Typically, the structure of a neural network may be complex in order to achieve better performance. Although the model of robot manipulator is highly nonlinear, by introducing the priori information of the system model, the neural network can be optimized, i.e., the number of nodes in neural networks can be reduced effectively while maintaining the learning efficiency (Fontaine and Germain, 2001). Inspired by this, a series dynamic neural networks are proposed to realize robotic control in realtime (Zhang et al., 2004; Li et al., 2017; Yang et al., 2018b). Based on the idea of constraint-optimization, quadratic-programming approaches haven been introduced for kinematic control of redundant manipulators. The designed outer-loop controller is described as equality constraints, and objective functions are established to describe certain performance of the system. Using the learning and parallel calculation ability, dynamic neural networks are established to solve the quadratic-programming problem online. The kinematic control is thus achieved by ensuring the equality constraints, and the flexibility is used by optimizing the objective functions. On the other hand, these methods is capable of handling inequality constraints and model uncertainties (Zhang et al., 2018; Li et al., 2019; Xu et al., 2019b). In Cheng et al. (1993), the obstacle avoidance strategy is described as equality constraints, but the parameters of escape velocity is difficult to obtain. In Zhang and Wang (2004), Zhang et al. propose an inequality based method, in which the distance between the robot and obstacles are formulated as a group of distances from critical points and robot links. On this basis, an improved method is proposed by Guo et al. in Guo and Zhang (2012), which is capable of suppressing undesirable discontinuity in the original solutions.

Motivated by the above observations, in this paper, we proposed a novel obstacle avoidance strategy based on deep recurrent neural networks. By abstracting robot and obstacles as a set of critical points, the distances between the robot and obstacles are approximately described by a group of point-to-point distances. And the obstacle avoidance is realized by inequality constraint described by class-K functions. Then the obstacle avoidance problem is reformulated as a QP problem in the speed level, and a deep recurrent neural network is designed to solve the QP online. Numerical results show that the robot is capable of avoiding the obstacles while tracking the predefined trajectories. Before ending this section, the main contributions of this paper are summarized as below

- The proposed deep RNN based controller is able to achieve both path tracking and obstacle avoidance, at the same time, physical constraints such as angular joints and velocities are satisfied.
- In this paper, we propose a class-K function based obstacle avoidance strategy, which has a more general form of description than traditional linear escape velocity methods.
- By abstracting robots and obstacles into critical point sets respectively, the distance between the robot and the obstacle can be described in a simpler way. Besides, numerical results show that the control algorithm can realize the avoidance of static and dynamic obstacles.

## 2. Problem Formulation

### 2.1. Basic Description

When a redundant robot is controlled to track a particular trajectory in the cartesian space, the positional description of the end-effector can be formulated as:

(1) x=f(θ),

where *x* ∈ ℝ^*m*^ and θ ∈ ℝ^*n*^ are the end-effector′s positional vector and joint angles, respectively. In the velocity level, the kinematic mapping between ẋ and $\dot{\theta }$ can be described as:

(2) $$\dot{x}=J\left(\theta \right)\dot{\theta },$$

where *J*(θ) ∈ ℝ^*m*×*n*^ is the Jacobian matrix from the end-effector to joint space.

In engineering applications, obstacles are inevitable in the workspace of a robot manipulator. For example, robot manipulators usually work in a limited workspace restricted by fences, which are used to isolated robots from humans or other robots. This problem could be even more acute in tasks which requires collaboration of multiple robots. Let *C*_1_ be the set of all the points on a robot body, and *C*_2_ be the set of all the points on the obstacles, then the purpose of obstacle avoidance of a robot manipulator is to ensure *C*_1_ ∪ *C*_2_ = ∅ at all times. By introducing *d* as a safety distance between the robot and obstacles, the obstacle avoidance is reformulated as

(3) $$|O_{j}A_{i}|\;\geq d,\;\forall A_{i}\in C_{1},\forall O_{i}\in C_{2}.$$

where $|O_{j}A_{i}|=\sqrt{{\left(A_{i}-O_{j}\right)}^{\text{T}}\left(A_{i}-O_{j}\right)}$ is the Euclidean norm of the vector *A*_*i*_*O*_*j*_.

**Figure 1:**
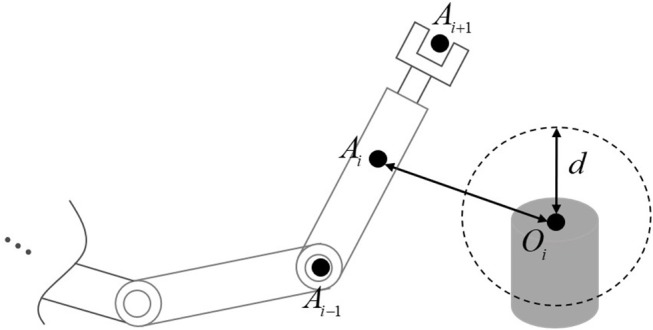
The basic idea of obstacle avoidance in this paper.

Equation (3) gives a basic description of obstacle avoidance problem in form of inequalities. However, there are too many elements in sets *C*_1_ and *C*_2_, the vast majority of which are actually unnecessary. Therefore, by uniformly selecting points of representative significance from *C*_1_ and *C*_2_, and increasing *d* properly, Equation (3) can be approximately described as below:

(4) $$|O_{j}A_{i}|\;\geq d,$$

with *A*_*i*_, *i* = 1, …, *a* and *O*_*j*_, *j* = 1, …, *b* being the representative points of the robot and obstacles, respectively. The schematic diagram of Equation (4) in shown in Figure 1.

*Remark. 1* In real implementations, there are many ways to measure |*O*_*j*_*A*_*i*_|. For instance, since physical structure of the a manipulator is known, the key points *A*_*i*_ are available in advance, both positions and velocities of those points can be calculated directly using the feedback of robot joints. The real-time measurement of obstacles can be achieved through industrial cameras. Therefore, the information of *A*_*i*_ and *B*_*j*_ are all available. As to measurement noise, by introducing a bigger safety distance *d*, e.g., *d* = 1.5(*d*_1_ + *d*_2_), the safety can be ensured.

### 2.2. Reformulation of Inequality in Speed Level

In order to guarantee the inequality (4), by defining *D* = |*O*_*j*_*A*_*i*_| − *d*, an inequality is rebuilt in speed level as:

(5) $$\text{d}\left(\text{|}O_{j}A_{i}\text{|}\right)\text{/d}t\geq -\text{sgn}\left(D\right)g\left(\text{|}D\text{|}\right),$$

in which *g*(•) belongs to class-*K*. Remarkable that the velocities of critical points *A*_*i*_ can be described by joint velocities:

(6) $${\dot{A}}_{i}=J_{ai}\left(\theta \right)\dot{\theta },$$

where $J_{ai}\in {\mathbb{R}}^{m\times n}$ is the Jacobian matrix from the critical point *A*_*i*_ to joint space. If *O*_*j*_ is prior known, the left-side of Equation (5) can be reformulated as:

(7) $$\begin{array}{l} \frac{\text{d}}{\text{d}t}\left(\left|O_{j}A_{i}\right|\right)=\frac{\text{d}}{\text{d}t}(\sqrt{{\left(A_{i}-O_{j}\right)}^{\text{T}}\left(A_{i}-O_{j}\right)})\\ \;=\frac{1}{\left|O_{j}A_{i}\right|}{\left(A_{i}-O_{j}\right)}^{\text{T}}\left({\dot{A}}_{i}-{\dot{O}}_{j}\right)\\ \;={\vec{\left|O_{j}A_{i}\right|}}^{\text{T}}J_{ai}(\theta )\dot{\theta }-{\vec{\left|O_{j}A_{i}\right|}}^{\text{T}}{\dot{O}}_{j}, \end{array}$$

where $\vec{\left|O_{j}A_{i}\right|}={\left(A_{i}-O_{j}\right)}^{\text{T}}/|O_{j}A_{i}|\in {\mathbb{R}}^{1\times m}$ is the unit vector of $\vec{A_{i}-O_{j}}$. Therefore, the collision between critical point *A*_*i*_ and object *O*_*j*_ can be obtained by ensuring the following inequality:

(8) $$J_{oi}\dot{\theta }\leq \text{sgn}\left(D\right)g\left(\left|D\right|\right)-{\vec{\left|O_{j}A_{i}\right|}}^{\text{T}}{\dot{O}}_{j},$$

where $J_{oi}=-{\vec{\left|O_{j}A_{i}\right|}}^{T}J_{ai}\in {\mathbb{R}}^{1\times n}$. Based on the inequality description (8), the collision between robot and obstacle can be avoided by ensuring:

(9) $$J_{o}\dot{\theta }\leq B,$$

where $J_{o}={\left[\underset{b}{\underset{︸}{J_{o1}^{\text{T}},\cdots \;,J_{o1}^{\text{T}}}},\cdots \;,\underset{b}{\underset{︸}{J_{oa}^{\text{T}},\cdots \;,J_{oa}^{\text{T}}}}\right]}^{\text{T}}\in {\mathbb{R}}^{ab\times n}$ is the concatenated form of *J*_*oi*_ considering all pairs between *A*_*i*_ and *O*_*j*_, $B={\left[B_{11},\cdots \;,B_{1b},\cdots \;,B_{a1},\cdots \;,B_{ab}\right]}^{\text{T}}\in {\mathbb{R}}^{ab}$ is the vector of upper-bounds, in which $B_{ij}=\text{sgn}(D)g(|D|)-{\vec{\left|O_{j}A_{i}\right|}}^{T}{\dot{\text{O}}}_{j}$.

*Remark 2:* According to 5 the definition of class-K functions, the original escape velocity based obstacle avoidance methods in Zhang and Wang (2004); Guo and Zhang (2012) can be regarded as a special case of 5, in which *G*(|*D*|) is selected as *G*(|*D*|) = *k*|*D*|. Therefore, in this paper, the proposed obstacle avoidance strategy is more general than traditional methods.

### 2.3. QP Type Problem Description

As to redundant manipulators, in order to take full advantage of the redundant DOFs, the robot can be designed to fulfill a secondary task when tracking a desired trajectory. In this paper, the secondary task is set to minimize joint velocity while avoiding obstacles. In real implementations, both joint angles and velocities are limited because of physical limitations such as mechanical constraints and actuator saturation. Because of the fact that rank(*J*) < *n*, there might be infinity solutions to achieve kinematic control. In this paper, we aim to design a kinematic controller which is capable of avoiding obstacles while tracking a pre-defined trajectory in the cartesian space. For safety′s sake, the robot is wished to move at a low speed, on the other hand, lower energy consumption is guaranteed. By defining an objective function scaling joint velocity as ${\dot{\theta }}^{\text{T}}\dot{\theta }/2$, the tracking control of a redundant manipulator while avoiding obstacles can be described as:

(10a) $$\text{min}\;{\dot{\theta }}^{\text{T}}\dot{\theta }/2,$$

(10b) $$s.t.\;x=x_{\text{d}},$$

(10c) $$\theta^{-}\leq \theta \leq \theta^{+},$$

(10d) $${\dot{\theta }}^{-}\leq \dot{\theta }\leq {\dot{\theta }}^{+},$$

(10e) $$J_{o}\dot{\theta }\leq B.$$

It is remarkable that the constraints 10b–10e and the objective function 10a to be optimized are built in different levels, which is very difficult to solve directly. Therefore, we will transform the original QP problem (10) in the velocity level. In order to realize precise tracking control to the desired trajectory *x*_d_, by introducing a negative feedback in the outer-loop, the equality constraint can be ensured by letting the end-effector moves at a velocity of ẋ = ẋ_d_ + *k*(*x*_d_ − *x*). In terms with (10c), according to escape velocity method, it can be obtained by limiting joint speed as $\alpha \left(\theta^{-}-\theta \right)\leq \dot{\theta }\leq \alpha \left(\theta^{+}-\theta \right)$, where α is a positive constant. Combing the kinematic property (2), the reformulated QP problem is:

(11a) $$\text{min}\;{\dot{\theta }}^{\text{T}}\dot{\theta }/2,$$

(11b) $$s.t.\;J\left(\theta \right)\dot{\theta }={\dot{x}}_{\text{d}}+k\left(x_{\text{d}}-x\right),$$

(11c) $$\text{max}\left(\alpha \left(\theta^{-}-\theta \right),{\dot{\theta }}^{-}\right)\leq \dot{\theta }\leq \text{min}\left({\dot{\theta }}^{+},\alpha \left(\theta^{+}-\theta \right)\right),$$

(11d) $$J_{o}\dot{\theta }\leq B.$$

It is noteworthy that both the formula (11a) and (11d) are nonlinear. The QP problem cannot be solved directly by traditional methods. Using the parallel computing and learning ability, a deep RNN will be established later.

## 3. Deep RNN Based Solver Design

In this section, a deep RNN is proposed to solve the obstacle avoidance problem (11) online. To ensure the constraints (11b), (11c), and (11d), a group of state variables are introduced in the deep RNN. The stability is also proved by Lyapunov theory.

### 3.1. Deep RNN Design

Firstly, by defining a group of state variables $\lambda_{1}\in {\mathbb{R}}^{m}$, $\lambda_{2}\in {\mathbb{R}}^{ab}$, a Lagrange function is selected as:

(12) $$L={\dot{\theta }}^{\text{T}}\dot{\theta }/2+\lambda_{1}^{\text{T}}\left({\dot{x}}_{\text{d}}+k\left(x_{\text{d}}-x\right)-J\left(\theta \right)\dot{\theta }\right)+\lambda_{2}^{\text{T}}\left(J_{o}\dot{\theta }-B\right),$$

λ_1_ and λ_2_ are the dual variables corresponding to the constraints (11b) and (11d). According to Karush-Kuhn-Tucker conditions, the optimal solution of the optimization problem (12) can be equivalently formulated as:

(13a) $$\dot{\theta }=P_{\Omega }(\dot{\theta }-\frac{\partial L}{\partial \dot{\theta }}),$$

(13b) $$J\left(\theta \right)\dot{\theta }={\dot{x}}_{\text{d}}+k\left(x_{\text{d}}-x\right),$$

(13c) $$\left\{ \begin{array}{l} \lambda_{2}>0\;\;\text{if}\;J_{o}\dot{\theta }=B,\\ \lambda_{2}=0\;\;\text{if}\;J_{o}\dot{\theta }\leq B, \end{array}\right.$$

where *P*_Ω_(*x*) = argmin_*y*∈Ω_||*y* − *x*|| is a projection operator to a restricted interval Ω, which is defined as $\Omega =\left\{\dot{\theta }\in {\mathbb{R}}^{n}|\text{max}\left(\alpha \left(\theta^{-}-\theta \right),{\dot{\theta }}^{-}\right)\leq \dot{\theta }\leq \text{min}\left({\dot{\theta }}^{+},\alpha \left(\theta^{+}-\theta \right)\right)\right\}$. Notable that Equation (13c) can be simply described as:

(14) $$\lambda_{2}={\left(\lambda_{2}+J_{o}\dot{\theta }-B\right)}^{+},$$

where (•)^+^ is a projection operation to the non-negative space, in the sense that *y*^+^ = max(*y*, 0).

Although the solution of (13) is exact the optimal solution of the constrained-optimization problem (11), it is still a challenging issue to solve (13) online since its inherent nonlinearity. In this paper, in order to solve (13), a deep recurrent neural network is designed as:

(15a) $$\epsilon \ddot{\theta }=-\dot{\theta }+P_{\Omega }\left(J^{\text{T}}\lambda_{1}-J_{o}^{\text{T}}\lambda_{2}\right),$$

(15b) $$\epsilon {\dot{\lambda }}_{1}={\dot{x}}_{\text{d}}+k\left(x_{\text{d}}-x\right)-J\left(\theta \right)\dot{\theta },$$

(15c) $$\epsilon {\dot{\lambda }}_{2}=-\lambda_{2}+{\left(\lambda_{2}+J_{o}\dot{\theta }-B\right)}^{+},$$

with ϵ is a positive constant scaling the convergence of (15).

*Remark. 3* As to the established deep RNN (15), the first dynamic equation is also the output dynamics, which combines the dynamics of state variables λ_1_ and λ_2_, as well as the physical limitations including joint angles and velocities. State variable λ_1_ is used to ensure the equality constraint (11b), as shown in (15b), λ_1_ is updated according to the difference between reference speed ẋ_d_ + *k*(*x*_d_ − *x*) and actually speed $J\left(\theta \right)\dot{\theta }$. Similarly, λ_2_ is used to ensure the inequality constraint 11d, which will be further discussed later. It is remarkable that ϵ plays an important role in the convergence of the deep RNN. The smaller ϵ, the faster the deep RNN converges.

*Remark. 4* By introducing the model information such as *J*, *J*_*o*_, etc., the established deep RNN consists of three class of nodes, namely $\dot{\theta }$, λ_1_ and λ_2_, and the total number of nodes is *n*+*m*+*ab*. Comparing to traditional neural networks in Jung and Kim (2007), the complexity of neural networks is greatly reduced.

### 3.2. Stability Analysis

In this subsection, we offer stability analysis of the obstacle avoidance method based on deep RNN based. First of all, some basic Lemmas are given as below.

*Definition 1:* A continuously differentiable function *F*(•) is said to be monotone, if ∇*F*+∇*F*^T^ is positive semi-definite, where ∇*F* is the gradient of *F*(•).

*Lemma 1:* A dynamic neural network is said to converge to the equilibrium point if it satisfies:

(16) $$\kappa \dot{x}=-x+P_{S}\left(x-\varrho F\left(x\right)\right),$$

where κ > 0 and ϱ > 0 are constant parameters, and *P*_*S*_ = argmin_*y*∈*S*_||*y* − *x*|| is a projection operator to closed set *S*.

*Lemma 2:* (Slotine and Li, 2004) Let *V* :[0,∞) × *B*_*d*_ → ℝ be a *C*^1^ function, α_1_, α_2_ be class-K functions defined on [0, *d*) which satisfy α_1_(||*x*||) ≤ *V*(*t, x*) ≤ α_2_(||*x*||), ∀(*t, x*) ∈ [0, *d*) × *B*_*d*_, then *x* = 0 is a uniformly asymptotically stable equilibrium point if there exist some class-K function *g* defined on [0, *d*), to make the following inequality hold:

(17) $$\frac{\partial V}{\partial t}+\frac{\partial V}{\partial x}f\left(t,x\right)\leq -\alpha \left(\left||x|\right|\right),\forall \left(t,x\right)\in [0,\infty )\times B_{d},$$

About the stability of the closed-loop system, we offer the following theorem.

*Theorem 1:* Given the obstacle avoidance problem for a redundant manipulator in kinematic control tasks, the proposed deep recurrent neural network is stable and will globally converge to the optimal solution of (10).

*Proof:* The stability analysis consists of two parts: firstly, we will show that the equilibrium of the deep RNN is exactly the optimal solution of the control objective described in (11), which prove that the output of deep RNN will realize obstacle avoidance while tracking a given trajectory, and then we will prove that the deep recurrent neural network is stable in sense of Lyapunov.

*Part I*. As to the deep recurrent neural network (15), let $\left({\dot{\theta }}^{*},\lambda_{1}^{*},\lambda_{2}^{*}\right)$ be the equilibrium of the deep RNN, then $\left({\dot{\theta }}^{*},\lambda_{1}^{*},\lambda_{2}^{*}\right)$ satisfies:

(18a) $$-{\dot{\theta }}^{*}+P_{\Omega }\left(J^{\text{T}}\lambda_{1}^{*}-J_{o}^{\text{T}}\lambda_{2}^{*}\right)=0,$$

(18b) $${\dot{x}}_{\text{d}}+k\left(x_{\text{d}}-x\right)-J\left(\theta \right){\dot{\theta }}^{*}=0,$$

(18c) $$-\lambda_{2}^{*}+{\left(\lambda_{2}^{*}+J_{o}{\dot{\theta }}^{*}-B\right)}^{+}=0,$$

with ${\dot{\theta }}^{*}$ be the output of deep RNN. By comparing Equation (18) and (13), we can readily obtain that they are identical, i.e., the equilibrium point satisfies the KKT condition of (10).

Then we will show that the equilibrium point(output of the proposed deep RNN) is capable of dealing with kinematic tracking as well as obstacle avoidance problem. Define a Lyapunov function *V* about the tracking error *e* = *x*_d_ − *x* as *V* = *e*^T^*e*/2, by differentiating *V* with respect to time and combining (11b), we have:

(19) $$\begin{array}{l} \dot{V}=e^{\text{T}}\dot{e}=e^{\text{T}}\left({\dot{x}}_{\text{d}}-J\left(\theta \right){\dot{\theta }}^{*}\right)\\ \;=-ke^{\text{T}}e\leq 0, \end{array}$$

in which the dynamic Equation 18b is also used. It can readily obtained that the tracking error would eventually converge to 0.

It is notable that the dynamic (Equation 18c) satisfies:

(20) $$\lambda_{2}^{*}+J_{o}{\dot{\theta }}^{*}-B-{\left(\lambda_{2}^{*}+J_{o}{\dot{\theta }}^{*}-B\right)}^{+}=J_{o}{\dot{\theta }}^{*}-B.$$

According to the property of projection operator (•)^+^, *y*−(*y*)^+^ ≤ 0 holds for any *y*, then we have $J_{o}{\dot{\theta }}^{*}-B\leq 0$, together with (5), the inequality (5) is satisfied. Notable that (5) can be rewritten as:

(21) $$\dot{D}\geq -\text{sgn}\left(D\right)g\left(\left|D\right|\right).$$

As to (21), we first consider the situation when equality holds. Since *g*(|*D*|) is a function belonging to class K, it can be easily obtained that *D* = 0 is the only equilibrium of Ḋ = −sgn(*D*)*g*(|*D*|). Define a Lyapunov function as $V_{2}\left(t,D\right)=D^{2}/2$, and select two functions as $\alpha_{1}\left(\left|D\right|\right)=\alpha_{2}\left(\left|D\right|\right)=D^{2}/2$. It is obvious that α_1_(|*D*|) = α_2_(|*D*|) belong to class-K, and the following inequality will always hold:

(22) $$\alpha_{1}\left(\left|D\right|\right)\leq V_{2}\left(t,D\right)\leq \alpha_{2}\left(\left|D\right|\right).$$

Taking the time derivative of *V*_2_(*t, D*), we have:

(23) $$\frac{\partial V_{2}}{\partial \text{t}}+\frac{\partial V}{\partial D}\dot{D}=-|D|g\left(\left|D\right|\right)\leq 0.$$

According to Lemma 2, the equilibrium *x* = 0 is uniformly asymptotically stable. Then we arrive at the conclusion that if the equality d(|*O*_*j*_*A*_*i*_|)/d*t* = −sgn(*D*)*g*(|*D*|) holds, |*D*| = 0 will be guaranteed, i.e., |*O*_*j*_*A*_*i*_| − *d* for all *i* = 1⋯*a*, = 1⋯*b*. Based on comparison principle, we can readily obtain that |*O*_*j*_*A*_*i*_| ≥ *d* when d(|*O*_*j*_*A*_*i*_|)/d*t* ≥ −sgn(*D*)*g*(|*D*|).

*Part II*. Then we will show the stability of the deep RNN (15). Let $\xi ={\left[{\dot{\theta }}^{\text{T}},\lambda_{1}^{\text{T}},\lambda_{2}^{\text{T}}\right]}^{\text{T}}$ be the a concatenated vector of state variables of the proposed deep RNN, then (15) can be rewritten as:

(24) $$\epsilon \dot{\xi }=-\xi +P_{\bar{\Omega }}[\xi -F\left(\xi \right)],$$

where *P*_*S*_(•) is a projection operator to a set *S*, and $F\left(\xi \right)={\left[F_{1}\left(\xi \right),F_{2}\left(\xi \right),F_{3}\left(\xi \right)\right]}^{\text{T}}\in {\mathbb{R}}^{n+m+ab}$, in which:

$$\left[\begin{array}{c} F_{1}\\ F_{2}\\ F_{3} \end{array}\right]=\left[\begin{array}{c} \dot{\theta }-J^{\text{T}}\lambda_{1}+J_{o}^{\text{T}}\lambda_{2}\\ J\dot{\theta }-{\dot{x}}_{\text{d}}-k\left(x_{\text{d}}-x\right)\\ -J_{o}{\dot{\theta }}^{*}-B \end{array}\right]$$

Let ∇*F* = ∂*F*/∂ξ, we have:

(25) $$\nabla F\left(\xi \right)=\left[\begin{array}{ccc} I & -J^{\text{T}} & J_{o}^{\text{T}}\\ J & 0 & 0\\ -J_{o}^{\text{T}} & 0 & 0 \end{array}\right]$$

According to the definition of monotone function, we can readily obtain that *F*(ξ) is monotone. From the description of (24), the projection operator *P*_*S*_ can be formulated as *P*_*S*_ = [*P*_Ω_; *P*_*R*_; *P*_Λ_], in which *P*_Ω_ is defined in (13), *P*_*R*_ can be regarded as a projection operator of λ_1_ to *R*, with the upper and lower bounds being ±∞, and $P_{\Lambda }={\left(•\right)}^{+}$ is a special projection operator to closed set ${\mathbb{R}}_{+}^{ab}$. Therefore, *P*_*S*_ is a projection operator to closed set $\left[\Omega ;{\mathbb{R}}^{m};{\mathbb{R}}_{+}^{ab}\right]$. Based on Lemma 1, the proposed neural network (15) is stable and will globally converge to the optimal solution of (10). The proof is completed.

## 4. Numerical Results

In this section, the proposed deep RNN based controller is applied on a planar 4-DOF robot. Firstly, a basic case where the obstacle is described as a single point is discussed, and then the controller is expanded to multiple obstacles and dynamic ones. Comparisons with existing methods are also listed to indicate the superiority of the deep RNN based scheme.

### 4.1. Simulation Setup

**Figure 2:**
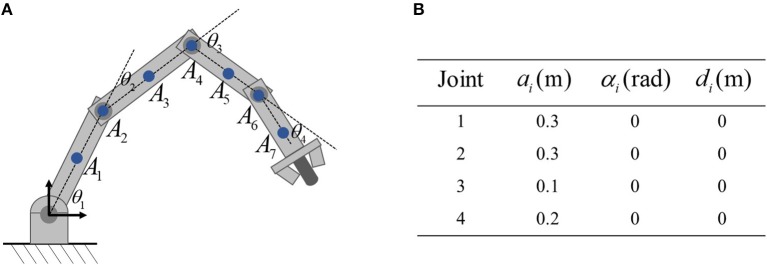
The planar robot to be simulated in this paper. **(A)** is the physical structure and critical points, **(B)** is the corresponding Dh parameters.

The physical structure of the 4-link planar robot to be simulated in shown in Figure 2, in which the critical points of the robot are also marked. As shown in Figure 2A, critical points *A*_2_, *A*_4_, *A*_6_ are selected at the joint centers, and *A*_1_, *A*_3_, *A*_5_, *A*_7_ are selected at the center of robot links. The D-H parameters are given in Figure 2B. It is notable that *A*_*i*_ and the Jacobian matrix *J*_*o*_*i* are essential in the proposed control scheme. Based on the above description of *A*_*i*_, the D-H parameters of *A*_1_ is *a*_1_ = 0.15, *a*_2_ = *a*_3_ = 0, α_1_ = α_2_ = α_3_ = 0, *d*_1_ = *d*_2_ = *d*_3_ = 0, then both the position and Jacobian matrix *J*_*a*1_ of *A*_1_ can be calculated readily. Based on the definition in Equation 8, *J*_*o*1_ can be obtained. *A*_*i*_ and *J*_*oi*_ can be calculated similarly. The control parameters are set as ϵ = 0.001, α = 8, *k* = 8. As to the physical constraints, the limits of joint angles and velocities are selected as $\theta_{i}^{-}=-3$rad, $\theta_{i}^{+}=3$rad, ${\dot{\theta }}_{i}^{-}=-1$rad/s, ${\dot{\theta }}_{i}^{+}=1$rad/s for *i* = 1…4. The safety distance *d* is set to be 0.1m.

### 4.2. Single Obstacle Avoidance

**Figure 3:**
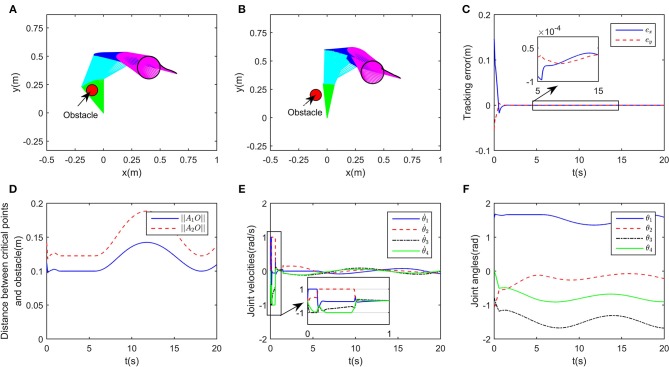
Numerical results of single obstacle avoidance. **(A)** is the motion trajectories when ignoring obstacle avoidance scheme, **(B)** is the motion trajectories when considering obstacle avoidance scheme, **(C)** is the profile of tracking errors when considering obstacle avoidance scheme, **(D)** is the profile of distances between critical points and obstacle, **(E)** is the profile of joint velocities, **(F)** is the profile of joint angles.

In this simulation, the obstacle is assumed to be centered at [−0.1, 0.2]^T^m, the desired path is set as $x_{\text{d}}={\left[0.4+0.1cos\left(0.5t\right),0.4+0.1sin\left(0.5t\right)\right]}^{\text{T}}$m, and the initial joint angles are set to be $\theta_{0}={\left[\pi /2,-\pi /3,-\pi /4,0\right]}^{\text{T}}$rad. The class-K function is selected as *G*(|*D*|) = *K*_1_|*D*| with *K*_1_ = 200. In order to show the effectiveness of the proposed obstacle avoidance method, contrast simulations with and without inequality constraint (10e) are conducted. Simulation results are shown in Figure 3. When ignoring the obstacle, the end-effector trajectories and the corresponding incremental configurations are shown in Figure 3A, although the robot achieves task space tracking to *x*_d_, obviously the first link of the robot would collide with the obstacle. After introducing obstacle avoidance scheme, the robot moves other joints rather than the first joint, and then avoids the obstacle effectively (Figure 3B). Simultaneously, the tracking errors when tracking the given circle are shown in Figure 3C. From the initial state, the end-effector moves toward the circle quickly and smoothly, after that, the tracking error in stable state keeps <1 × 10^−4^m, showing that the robot could achieve kinematic control as well as obstacle avoidance tasks. To show more details of the proposed deep RNN based method, some important process data is given. As the obstacle is close to the first joint, critical points *A*_1_ and *A*_2_ are more likely to collide with obstacle, therefore, as a result, the distances between the obstacle *O*_1_ and *A*_1_, *A*_2_ are shown in Figure 3D, from *t* = 2s to *t* = 6.5s, ||*A*_1_*O*_1_|| remains at the minimum value *d* = 0.1, that is to say, using the proposed obstacle avoidance method, the robot maintains minimum distance from the obstacle. The profile of joint velocities are shown in Figure 3E, at the beginning of simulation, the robot moves at maximum speed, which leads to the fast convergence of tracking errors. The curve of joint angles change over time is shown in Figure 3F.

### 4.3. Discussion on Class-K Functions

**Figure 4:**
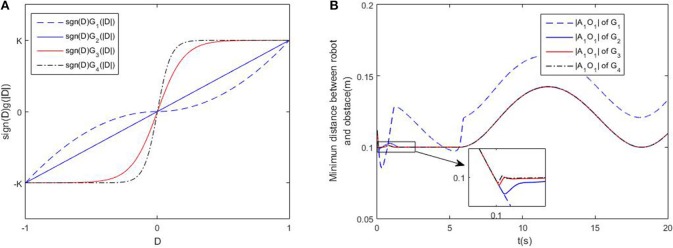
Discussions on different obstacle avoidance functions. **(A)** is the comparative curves of different obstacle avoidance functions. **(B)** is the profile of minimum distance of the robot and obstacle using different obstacle avoidance functions.

In this part, we will discuss the influence of different class-K functions in the avoidance scheme (5). Four functions are selected as $G_{1}\left(\left|D\right|\right)=K|D{|}^{2}$, *G*_2_(|*D*|) = *K*|*D*|, *G*_3_(|*D*|) = *K*tanh(5|*D*|), *G*_4_(|*D*|) = *K*tanh(10|*D*|), Figure 4A shows the comparative curves the these functions. Other simulation settings are the same as the previous one. Simulation results are shown in Figure 4B. When selecting the same positive gain *K*, the minimum distance is about 0.08m, which shows the robot can avoid colliding with the obstacle using the avoidance scheme (5). The close-up graph of the tracking error is also shown, it is remarkable that the minimum distance deceases, as the gradient of the class-K function increases near 0. Therefore, one conclusion can be drawn that the function can be more similar with Sign function, to achieve better obstacle avoidance.

### 4.4. Multiple Obstacles Avoidance

**Figure 5:**
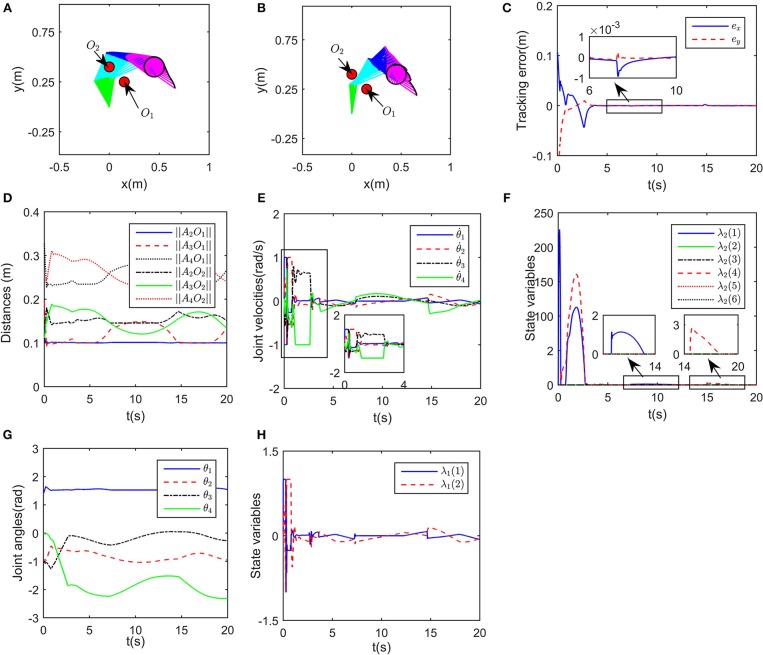
Numerical results of multiple obstacle avoidance. **(A)** is the motion trajectories when ignoring obstacle avoidance scheme, **(B)** is the motion trajectories when considering obstacle avoidance scheme, **(C)** is the profile of tracking errors when considering obstacle avoidance scheme, **(D)** is the profile of distances between critical points and obstacles, **(E)** is the profile of joint velocities, **(F)** is the profile of λ_2_, **(G)** is the profile of joint angles, **(H)** is the profile of λ_1_.

In this part, we consider the case where there are two obstacles in the workspace. The obstacles are set at [0.1, 0.25]^T^m and [0, 0.4]^T^m, respectively. Simulation results are shown in Figure 5. The desired path is defined as $x_{\text{d}}={\left[0.45+0.1cos\left(0.5t\right),0.4+0.1sin\left(0.5t\right)\right]}^{\text{T}}$. The initial joint angle of the robot is selected as $\theta_{0}={\left[1.5,-1-1,0\right]}^{\text{T}}$. To further show the effectiveness of the proposed obstacle avoidance strategy 5, *g*|*D*| is selected as $g|D|=K_{1}/\left(1+e^{-|D|}\right)-K_{1}/2$ with *K*_1_ = 200. When λ_2_ is set to 0, as shown in Figure 5A, the inequality constraint (11d) will not work, in other words, only kinematic tracking problem in considered rather than obstacle avoidance, in this case, the robot would collide with the obstacles. After introducing online training of λ_2_, the simulation results are given in Figures 5B–H. The tracking errors are shown in Figure 5C, with the transient time being about 4s, and steady state error <1 × 10^−3^m. Correspondingly, the robot moves fast in the transient stage, ensuring the quick convergence of the tracking errors. It is remarkable that the distances between the critical points and obstacle points are kept larger than 0.1m at all times, showing the effectiveness of the proposed method. At *t* = 14s, from Figures 5D,G, when the distance between *A*_3_ and *O*_1_ is close to 0.1m, the corresponding dual variable λ_2_ becomes positive, making the inequality constraint (11d) hold, and the boundedness between the robot and obstacle is thus guaranteed. After *t* = 18s, ||*A*_3_*O*_1_|| becomes greater, then λ_2_ converges to 0. Notable that although λ_1_ and λ_2_ do not converge to certain values, the dynamic change of λ_1_ and λ_2_ ensures the regulation of the proposed deep RNN.

### 4.5. Enveloping Shape Obstacles

**Figure 6:**
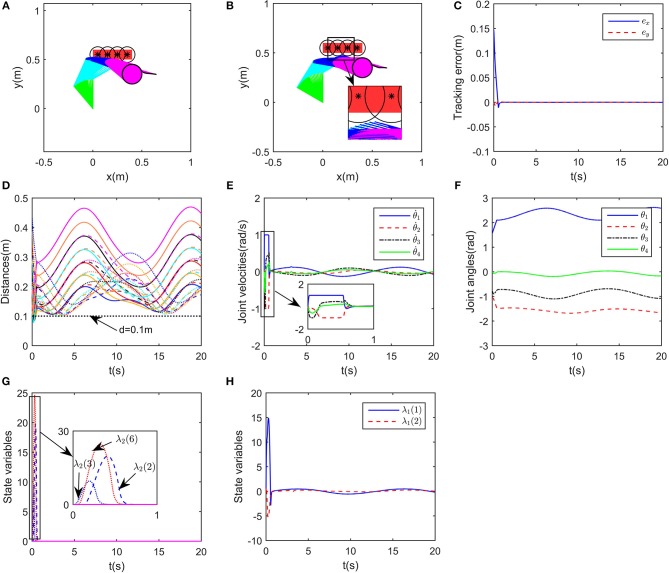
Numerical results of enveloping shape obstacles. **(A)** is the motion trajectories when ignoring obstacle avoidance scheme, **(B)** is the motion trajectories when considering obstacle avoidance scheme, **(C)** is the profile of tracking errors when considering obstacle avoidance scheme, **(D)** is the profile of distances between critical points and obstacles, **(E)** is the profile of joint velocities, **(F)** is the profile of joint angles, **(G)** is the profile of λ_2_, **(H)** is the profile of λ_1_.

In this part, we consider obstacles of general significance. Suppose that there is a rectangular obstacle in the workspace, with the vertices being [0, 0.5]^T^, [0.4, 0.5]^T^, [0.4, 0.6]^T^ and [0.5, 0.6]^T^, respectively. By selecting the safety distance *d* = 0.1m, and obstacle points as $O_{1}={\left[0.05,0.55\right]}^{\text{T}}$, $O_{2}={\left[0.15,0.55\right]}^{\text{T}}$, $O_{3}={\left[0.25,0.55\right]}^{\text{T}}$ and $O_{4}={\left[0.35,0.55\right]}^{\text{T}}$. It can be readily obtained that the rectangular obstacle is totally within the envelope defined by *O*_*i*_ and *d*. The incremental configurations when tracking the path while avoiding the obstacle are shown in Figure 6B, in which a local amplification diagram is also given. showing that the critical points *A*_3_ is capable of avoiding *O*_2_ and *O*_3_. It is worth noting that by selecting proper point group and safety distance, the obstacle can be described by the envelope shape effectively. While in Figure 6A, when obstacle avoidance is ignored, the collision emerges. Figures 6C–H also give important process data of the system under the proposed controller, including tracking errors, joint angles, angular velocities, and state variables of deep RNNs. We can observe that the physical constraints as well as kinematic control task are realized using the controller.

### 4.6. Dynamic Obstacles

**Figure 7:**
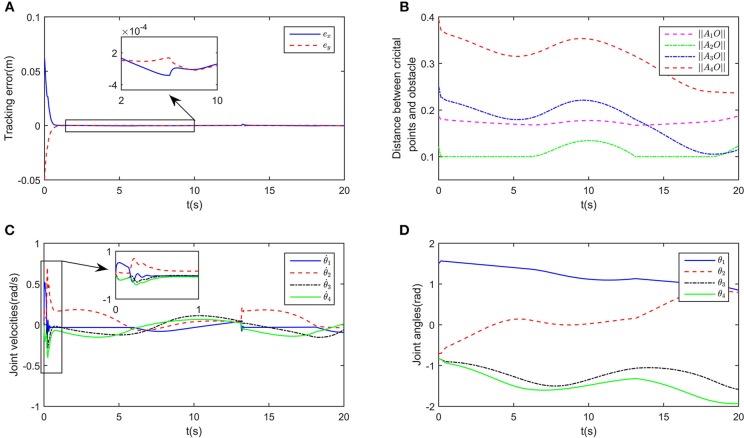
Numerical results of enveloping shape obstacles. **(A)** is the profile of tracking errors when considering obstacle avoidance scheme, **(B)** is the profile of distances between critical points and obstacles, **(C)** is the profile of joint velocities, **(D)** is the profile of joint angles.

**Figure 8:**
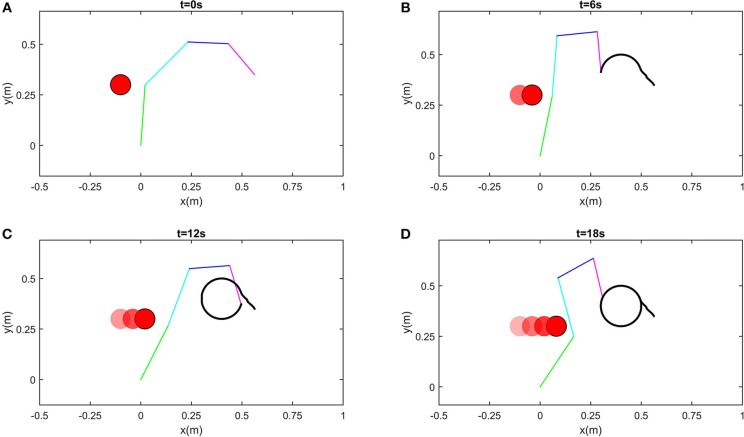
Snapshots when robot avoiding a dynamic obstacle. **(A)** is the snapshot when *t* = 0s, **(B)** is the snapshot when *t* = 6s, **(C)** is the snapshot when *t* = 12s, **(D)** is the snapshot when *t* = 18s.

In this part, we consider dynamic obstacles moving in the workspace. In real applications, pedestrian or other obstacles always tend to be mobile. Obstacle avoidance for dynamic obstacles is of more general significance. In real time, static obstacles can be considered a special case. In this simulation, the simulation duration is selected as 20s, and the trajectory of a dynamic obstacle is defined as $x_{\text{d}}={\left[-0.1+0.01t,0.3\right]}^{\text{T}}$. The snapshots in the control process are shown in Figure 8. While ensuring effective tracking of the defined path, the robot is able to use its self-motion to avoid the dynamic obstacle effectively, and maintain a safe distance. The distances between critical points and the dynamic *O* is shown in Figure 7B. At the beginning of simulation, the tracking error is big, in order to ensure the convergence of tracking error, the joints move a big range except *J*1. It is worth noting that since the critical point *A*_2_ is next to *O*, joint 1 rotates in the direction which conforms to the movement of *O*. In the stable state, tracking error is < 5 × 10^−4^m (Figure 7A). At about *t* = 14s, *A*_2_*O* decreases to 0.1m, accordingly, the joint velocities change obviously (as shown the significant change of joint velocities in Figure 7C, the tracking error changes to 10^−3^m, and then converges quickly. At *t* = 18s, although *A*_2_ and *A*_3_ are near *O*, the robot is still capable of avoiding the dynamic obstacle. During the control process, joint angles are ensured not to exceed the limits, as shown in Figure 7D.

### 4.7. Obstacle Performance on 7-DOF Manipulator in 3-Dimensional Space

**Figure 9:**
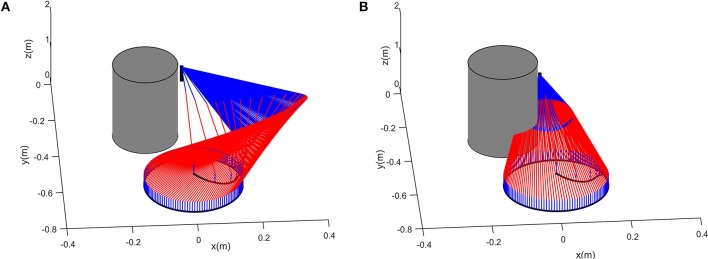
Comparative results when the proposed controller is used on a 7-DOF manipulator iiwa in 3-dimensional space. **(A)** is the tracking trajectory and the corresponding joint configurations when obstacle avoidance scheme is introduced. **(B)** is the tracking trajectory and the corresponding joint configurations when obstacle avoidance scheme is not introduced.

To further verify the effectiveness of the control scheme, another simulation on a 7DOF manipulator iiwa is carried out. The desired path to be tracked is also a planar circular, which is centered at [0, −0.6, 0.1]^T^m with radius being 0.15m. The physical parameters can be found in Xu et al. (2019a). Suppose that there exist a cylinder obstacle in the workspace, the obstacle is centered as [−0.13, −0.3, 0]^T^m, with the radius and height being 0.15m and 2m, respectively. Simulation results are shown in Figure 9. It can be readily found that the proposed schemes can obtain satisfying performance in 3-dimensional spaces.

### 4.8. Comparisons

To illustrate the priority of the proposed scheme, a group of comparisons are carried out. As shown in Table 1, all the controllers in Zhang and Wang (2004); Csiszar et al. (2011); Guo and Zhang (2012); Krzysztof and Joanna (2016) achieve the avoidance of obstacles. Comparing to APF method in Csiszar et al. (2011); Krzysztof and Joanna (2016) of JP based method in Csiszar et al. (2011); Krzysztof and Joanna (2016), the proposed controller can realize a secondary task, at the same time, we present a more general formulation of the obstacle avoidance strategy, which is helpful to gain a deeper understanding of the mechanism for avoidance of obstacles. Moreover, in this paper, both dynamic trajectories and obstacles are considered. The comparisons above also highlight the main contributions of this paper.

**Table 1:** Comparisons among different obstacle avoidance controllers on manipulators.

**Method**	**Convergence**	**Secondary task**	**Handling physical constraints**	**Dynamic obstacles**	**obstacle avoidance description**
This paper	Yes	Yes	Yes	Yes	Inequalities
Guo and Zhang, 2012	Yes	Yes	Yes	^*^	Inequalities^**^
Zhang and Wang, 2004	Yes	Yes	Yes	^*^	Inequalities^**^
Csiszar et al., 2011	Yes	No	No	Yes	Repulsion
Krzysztof and Joanna, 2016	Yes	No	No	^*^	Null space

* *In Zhang and Wang (2004); Guo and Zhang (2012); Krzysztof and Joanna (2016), dynamic obstacles are not considered*.

** *Regular escape velocity method is used, which is only a special case of 5*.

## 5. Conclusions

In this paper, a novel obstacle avoidance strategy is proposed based on a deep recurrent neural network. The robots are obstacles are presented by sets of critical points, then the distance between the robot and obstacle can be approximately describes as point-to-points distances. By understanding the nature escape velocity methods, a more general description of obstacle avoidance strategy is proposed. Using minimum-velocity-norm (MVN) scheme, the obstacle avoidance together with path tracking problem is formulated as a QP problem, in which physical limits are also considered. By introducing model information, a deep RNN with simple structure is established to solve the QP problem online. Simulation results show that the proposed method can realize the avoidance of static and dynamic obstacles.

## Data Availability

All datasets analyzed for this study are included in the manuscript and the supplementary files.

## Author Contributions

Theoretical derivation was done by ZX. Simulation research was performed by ZX, with important advice from XZ. The paper was revised by XZ and SL. All authors approved the manuscript.

### Conflict of Interest Statement

The authors declare that the research was conducted in the absence of any commercial or financial relationships that could be construed as a potential conflict of interest.
